# Retrieval-related Eye Movements Are Predictive of Memory Precision

**DOI:** 10.1162/JOCN.a.2627

**Published:** 2026-10-01

**Authors:** Mingzhu Hou, Luke R. Pezanko, Sabina Srokova, Paul F. Hill, Arne D. Ekstrom, Michael D. Rugg

**Affiliations:** The University of Texas at Dallas; University of Arizona

## Abstract

Eye movements are predictive of successful episodic memory encoding and retrieval, but it is unclear whether they reflect the precision of retrieved memory content. Here, we examined relationships between eye fixations, memory precision, and fMRI BOLD activity. At study, participants were presented with object images, each placed at a random location on a circle. At test, both studied and new images were presented. Participants were instructed to recall and signal the studied location of each image, guessing if necessary. They then indicated if the image had been studied. Simultaneous fMRI and eye-tracking data were acquired during the test phase. For correctly recognized images, trial-wise angular distances between the studied and reported locations were fitted to a two-component mixture model. On the basis of model-derived parameters, correctly recognized images were categorized as those associated with successful location retrieval (location hits) or guesses. Location hits were further divided into high- and low-precision trials. At retrieval, fixation location predicted both successful retrieval and the precision of the retrieved location memory. In addition, across-trial fixation patterns were more similar for location hits than for guesses and for high- than for low-precision trials. The association between retrieval success and fixation precision was evident across almost the entire recall phase, whereas memory precision effects emerged around 1–1.5 sec after image onset. Fixation precision and pattern similarity each predicted trial-wise memory precision independently of hippocampal BOLD activity, which also predicted memory precision. Taken together, the findings indicate that eye movements during retrieval track the fidelity of retrieved mnemonic content.

## INTRODUCTION

Episodic memory is mainly assessed through overt memory judgments, but analysis of incidental eye movements can provide additional insight into how episodic memories are encoded and retrieved. Notably, fixation patterns during both the study and test phases of episodic memory tasks have been reported to reflect mnemonic content (for reviews, see Ryan & Shen, 2020; Wynn, Shen, & Ryan, 2019). Here, we focus on the relationship between fixation patterns and memory performance at retrieval.

Prior findings provide evidence for an association between fixation patterns and memory retrieval (e.g., Damiano & Walther, 2019; Johansson & Johansson, 2014; Olsen, Chiew, Buchsbaum, & Ryan, 2014; Hannula & Ranganath, 2009; for reviews, see Ryan & Shen, 2020; Hannula et al., 2010). For example, in a study examining object-location memory, Johansson and Johansson (2014) reported that, in a free-viewing condition, fixations were more likely to be directed toward the quadrant of the display where object images had been studied. By contrast, constraining eye movements to a different location impaired memory for the object. The findings from this and other studies cited above indicate that fixation patterns during retrieval predict successful memory retrieval.

Other recent studies have examined associations between anticipatory gaze and retrieved mnemonic content. For instance, Schmidig and colleagues (2025) reported that when participants rewatched animated movie clips containing a surprising event, they made anticipatory fixations toward the location of the event before its occurrence. Furthermore, anticipatory fixations that were closer to the event's location on the display were associated with an increased likelihood of later identifying the quadrant of the screen in which the event occurred. Similarly, Büchel and colleagues (2024) had participants repeatedly learn a list of image exemplars belonging to one of five visual categories. Across repetitions, participants showed improved memory performance for the sector containing each exemplar and demonstrated anticipatory fixations that shifted closer to the exemplar's location.

Although findings from the aforementioned studies suggest that fixation patterns at retrieval vary according to the content of the retrieved memory, it is unclear whether fixations are predictive of memory precision. Addressing this question will shed light on the fidelity with which eye movements track retrieved memory content and their potential as an incidental marker of successful retrieval (cf. Schmidig et al., 2025). Recent studies examining memory precision have employed a positional response accuracy task (Harlow & Donaldson, 2013), in which memory performance is evaluated with a continuous metric that reflects the degree to which a feature of the study event can be accurately reproduced. Findings from behavioral studies employing positional response accuracy tasks suggest that retrieval success is dissociable from memory precision (e.g., Gellersen, McMaster, Abdurahman, & Simons, 2024; Harlow & Yonelinas, 2016; see also Kolarik, Baer, Shahlaie, Yonelinas, & Ekstrom, 2018, and Nilakantan, Bridge, VanHaerents, & Voss, 2018, for evidence from patients with hippocampal damage).

Several studies have employed fMRI to examine the neural correlates of retrieval success and precision, consistently implicating the hippocampus and left angular gyrus (AG) (Hou, Hill, Aktas, Ekstrom, & Rugg, 2025; Hou, Hill, Pezanko, et al., 2025; Korkki, Richter, Gellersen, & Simons, 2023; Cooper et al., 2017; Richter, Cooper, Bays, & Simons, 2016). In a recent study (Hou, Hill, Pezanko, et al., 2025), we examined the fMRI correlates of memory precision in a combined data set that included the same sample we report on below as well as a separate sample that was originally described by Hou, Hill, Aktas, and colleagues (2025). We reported that BOLD activity in the hippocampus and the left AG tracked trial-wise estimates of memory precision, whereas no effects of retrieval success could be identified in either region.

The present study had two primary aims. The first aim was to examine whether eye fixations predict the precision with which location information can be retrieved. Given prior evidence demonstrating robust associations between eye movements and neural activity in the hippocampus (for reviews, see Kragel & Voss, 2022; Ryan, Shen, & Liu, 2020), our second aim was to examine associations between memory precision, precision-related eye fixations, and hippocampal fMRI BOLD signals.

## METHODS

The behavioral and fMRI data pertaining to the present study sample were first reported as part of the data set described by Hou, Hill, Pezanko, and colleagues (2025). Here, we report the relationships between these data and eye-tracking metrics. These data have not been reported previously.

### Participants

Participants were 24 young adults (mean age = 23 years, age range = 18–33 years, 11 women). The sample size was consistent with that of prior fMRI studies examining the neural correlates of memory precision in young adults (Hou, Hill, Aktas, et al., 2025, *n* = 23; Korkki et al., 2023, *n* = 23; Richter et al., 2016, *n* = 21) and with prior studies examining the relationship between eye fixations and memory performance (e.g., Büchel et al., 2024, *n* = 27; Johansson & Johansson, 2014, *n* = 24). All participants were cognitively healthy and right-handed, had normal or corrected-to-normal vision, had no history of neurological or psychiatric illness, and were not taking prescription medications that affected the central nervous system.

Informed consent was obtained in accordance with the University of Texas at Dallas institutional review board guidelines. Participants were compensated at the rate of $30 an hour. Eye movement data from two participants were excluded because of poor calibration, defined as a validation error exceeding 1° for any fixation as based on EyeLink's default threshold. Accordingly, we report data from the remaining 22 participants.

### Experimental Items

**Figure 1.**
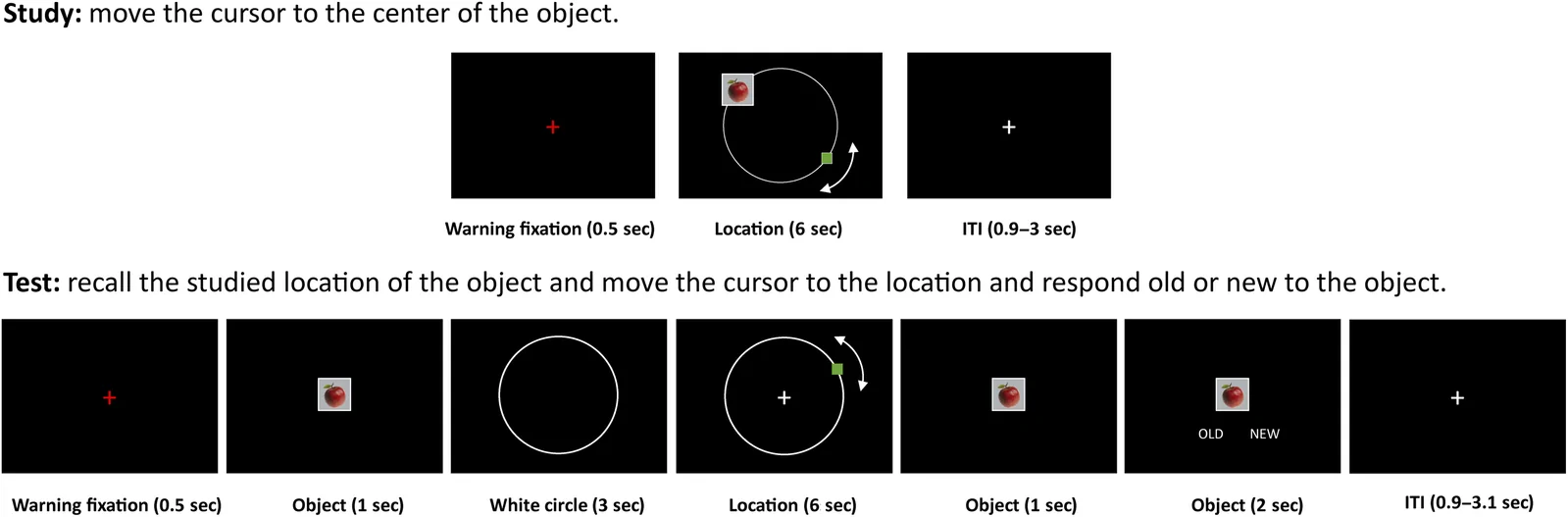
Schematics of the study and test phases. ITI = intertrial interval.

The experimental items were 136 images of everyday objects (e.g., apple, book, coin), which were randomly drawn from Hemera Photo Objects 50000 Volume III (www.hemera.com/index.html; see Figure 1). A total of 102 of these images were used as study items, and the remaining 34 images served as new (unstudied) test items.

### Procedure

The schematics for the experimental tasks are shown in Figure 1. There was a single study block followed by four consecutive test blocks. Both the study and test phases were undertaken in the MRI scanner.

At study, participants viewed 102 object images (visual angle = 1.4° × 1.4°, image size = 123 × 123 pixels), each presented for 6 sec at a random location on a circle (radius = 4.9° of visual angle, equivalent to 422 pixels). The study locations of the images were determined by randomly selecting values between 1° and 360° without replacement. Along with the image, a small green cursor was presented on the circle at a distance of at least 60° from the image. The instruction was to use a button box to move the cursor around the circle to the center of the image and to remember both the image and its location. A 30-sec break occurred in the middle of the study block, during which a “rest” cue was presented at the center of the screen. Participants were instructed to relax until the disappearance of the cue.

The test images comprised all of the studied images intermixed with 34 unstudied images. They were presented pseudorandomly so that no more than three studied or new images occurred in succession. Each test trial began with the presentation of the test image for 1 sec, after which a white circle of the same size as the virtual circle used during the study phase was presented for 3 sec. Participants were instructed to covertly recall the studied location of the image. They were then to move a cursor that was randomly located on the circle (with the constraint that it was no less than 60° from the image's studied location) to the recalled location, again by using a button box, guessing if necessary. After moving the cursor, participants signaled whether the presented object had been studied or was unstudied. Participants were not instructed to maintain fixation during the trial and hence were free to move their eyes.

Participants viewed the experimental stimuli via a mirror mounted on the scanner head coil that reflected a back-projected image (viewing distance: 102 cm) on a translucent screen (41 cm × 23 cm; resolution: 1920 × 1080 pixels). They pressed buttons under their right index and middle fingers to move the cursor along the circle during the study and test phases and to respond “old” or “new” to the test image. The mappings between the button presses and the cursor movement direction, as well as the old/new judgments, were counterbalanced across participants.

Eye movement data were acquired during the study and test phases with an MRI-compatible eye tracker (EyeLink 1000 Plus; SR Research). Monocular data were collected from the right eye at a sampling rate of 1000 Hz. Because drift in eye-tracking accuracy is minimal when calibration is conducted just before a scanner run (Peitek et al., 2018), calibration and manual validation were performed using EyeLink's 9-point calibration procedure immediately before each run. This procedure was repeated until the validation accuracy at each gaze point was acceptable according to EyeLink's default threshold (1°). We used EyeLink's default event parser to classify eye movements into saccades, blinks, and fixations. Saccades were defined by a velocity threshold of 30°/sec and an acceleration threshold of 8000°/s^2^. Samples with missing pupil size data were classified as blinks. The remaining data were categorized as fixations.

### Behavioral Analyses

#### Item Memory Performance

Item memory performance (Pr) was calculated as the difference between the proportion of item hits (studied objects that were correctly recognized) and the proportion of false alarms to new items.

#### Separation of Location Hits and Guess Trials

For each item hit trial, distance error was calculated as the angular difference between the reported location and the studied location. Distance errors for item hits were pooled across participants and fitted to a mixture model using the *MLE()* function implemented in MemToolbox (Suchow, Brady, Fougnie, & Alvarez, 2013). This procedure estimates a rectangular distribution of the proportion of guess responses (g), and a von Mises distribution of item hit trials associated with varying levels of memory precision (quantified by the concentration parameter [Kappa]).

Item hit trials were divided into successful (location hit) and unsuccessful (guess) trials depending on whether or not the associated distance errors had a < 5% chance (cf. Richter et al., 2016) of fitting the aforementioned across-participant von Mises distribution (estimated with the *vonmisescdf* function from www.paulbays.com/toolbox/). According to this criterion, the cutoff for distinguishing location hits from guess trials was ±36°. Therefore, location hits were defined as item hit trials with an absolute distance error < 36°. To minimize the likelihood of miscategorizing trials associated with successful retrieval as guesses, for the purposes of the analyses reported below, guess trials were defined as trials with an absolute distance error between 60° and 180° (cf. Hou, Hill, Aktas, et al., 2025; Hou, Hill, Pezanko, et al., 2025).

To facilitate the comparison of location hit trials that varied in precision, they were subdivided into high-precision and low-precision trials based on their associated absolute distance errors. High-precision trials were defined as location hits with an absolute distance error < 16°. Low-precision trials were defined as location hits with an absolute distance error ranging from 20° to 35°. The 4° gap between the two classes of trial was inserted to emphasize any distinctions between them in respect of their associated oculomotor or neural correlates (cf. Hou, Hill, Aktas, et al., 2025; Hou, Hill, Pezanko, et al., 2025).

### Analyses of Eye Fixation Data

#### Preprocessing of Fixation Data

Eye movement data were preprocessed using the *edfmex.m* function in MATLAB 2023a (Mathworks Inc., 2023). Fixations with a duration shorter than 50 msec were excluded from analyses (on average, 12% [*SD* = 9%] of the fixations from each participant were excluded based on this criterion). To differentiate image-related fixations from location-related fixations, a square was drawn at the center of the screen with sides twice the length of the test image. Location-related fixations (hereafter, fixations) were defined as those falling outside this central square.

#### Effects of Retrieval Success and Memory Precision on Fixation Metrics

In the following analyses, we derived trial-wise measures of the distance between fixation location and the location of the image at study (hereafter, fixation precision). In addition, we computed the similarity of retrieval-related fixation distribution patterns separately for location hits and guess trials (i.e., fixation pattern similarity; see below). In these analyses, retrieval success effects were examined by contrasting these fixation metrics between location hit and guess trials (note that the results were essentially the same when comparing only low-precision trials with guesses). Memory precision effects were identified by examining associations between trial-wise fixations metrics and absolute distance error for location hit trials.

#### The Effect of Retrieval Success on Fixation Precision

For a given item hit trial, fixation precision was operationalized as the mean Euclidean distance (in pixels) of each fixation relative to the studied location during the 4-sec interval extending from the onset of the test image to the end of the recall phase. We elected to employ Euclidean distance rather than angular error when estimating fixation precision to allow for the fact that fixations might be directed to locations above or below, as well as along, the circle on which the image was located at study. To assess differences in fixation precision between location hits and guess trials, we conducted a pairwise *t* test comparing the fixation precision metrics for the two classes of trial across participants.

#### The Effect of Memory Precision on Fixation Precision

To examine whether eye fixation metrics were sensitive to memory precision, we employed linear mixed-effects (LME) analyses using the *lmer()* function in R (R Core Team, 2018). An LME model was constructed to examine the relationship across location hits between trial-wise fixation precision and trial-wise judgment precision (measured by absolute distance error). The model employed fixation precision as a fixed effect predictor of trial-wise absolute distance error. Participants were modeled as a random intercept (LME Syntax: Memory Precision ∼ Fixation Precision + [1|Participant]).

#### Time Series Analyses

To examine the time course of the fixations associated with location hit and guess trials, we conducted a time-series analysis, in which the 4-sec recall phase was separated into eight successive 500-msec time bins. Fixation precision in each time bin was calculated as the precision of each fixation falling within the bin, weighted by its duration within that bin. An LME model was implemented with Trial Type (location hit, guess), Time Bin, and their interaction as the predictors of fixation precision. Participants were treated as a random intercept (LME Syntax: Fixation Precision ∼ Trial Type + Time Bin + Trial Type × Time Bin + [1|Participant]). A significant Trial Type × Time Bin interaction was followed up with an LME analysis employing trial type (location hit vs. guess) to predict fixation precision at each time bin (LME Syntax: Fixation Precision ∼ Trial Type + [1|Participant]). Because not all participants had fixations in all time bins, we implemented an LME approach rather than a participant-wise ANOVA because it is more applicable to the analysis of unbalanced data. We conducted an analogous analysis to compare the time courses of fixations associated with high- versus low-precision location hits. In both sets of analyses, significant time bins were defined as those that survived false discovery rate (FDR) correction.

To examine whether any potential effect of trial type was associated with differences in prestimulus fixation patterns, we additionally analyzed fixation precision for the 500-msec time bin immediately before the onset of the test image (see Figure 1).

#### Pattern Similarity Analyses

To examine whether the spatial patterning of eye fixations varied with trial type, we conducted pattern similarity analyses on the fixation data. Because the studied locations were unique across the item hit trials, the locations were realigned to a common reference point (the top point of the virtual circle; see Figure 5). Coordinates of fixations within each trial were adjusted accordingly to preserve their relative distance to the studied location.

Pattern similarity analyses were performed using the eyesim package (Buchsbaum, 2025). For each participant, trial-wise fixation density maps were generated by applying a duration-weighted Gaussian smoothing kernel (sigma = 50 pixels) to the adjusted fixations (the results were essentially unchanged when the analyses were repeated with kernel sizes of 25 or 100 pixels). The density map for each trial was then vectorized by flattening the density values from all cells into a single column vector.

For each participant, we computed trial-wise pattern similarity metrics for location hits and guesses. For each location hit trial, pattern similarity was computed as the Fisher *z-*transformed correlation between its density map and the maps corresponding to all other location hit trials, minus the mean Fisher *z-*transformed correlation between its associated density map and the maps corresponding to all guess trials. An analogous similarity metric was computed for guess trials. We employed one-sample *t* tests to test the similarity metrics associated with different trial types against the null hypothesis of zero similarity. Pairwise *t* tests were conducted to compare the participant-wise similarity metrics between location hits and guesses and between high- and low-precision trials.

#### Control Analysis

We examined whether eye fixations were merely a predictor of the location of the upcoming manual judgment (i.e., cursor placement) independent of the accuracy of the retrieved mnemonic content. This was achieved by repeating the aforementioned fixation precision and pattern similarity analyses relative to the location signaled by the participants (i.e., judged location) rather than the studied location. We compared the resulting “fixation precision” metric between location hit and guess trials, and separately between high and low precision trials. Similarity metrics for the different trial types were contrasted against a null hypothesis of zero. The metrics were also contrasted between location hit and guess trials and between high- and low-precision trials.

### MRI Acquisition and Preprocessing

As reported in more detail previously (Hou, Hill, Pezanko, et al., 2025), MRI data were acquired with a Siemens PRISMA 3-T MR scanner equipped with a 32-channel head coil. Functional images were acquired with a T2^⁎^-weighted echo-planar sequence (repetition time [TR] = 1560 msec, echo time [TE] = 30 msec, flip angle = 70°, field of view [FOV] = 220 mm, multiband factor = 2, 48 slices, voxel size = 2.5 × 2.5 × 2.5 mm, 0.5-mm interslice gap, anterior-to-posterior phase encoding direction). A T1-weighted image was acquired with a magnetization prepared rapid acquisition gradient echo pulse sequence (TR = 2300 msec, TE = 2.41 msec, FOV = 256 mm, voxel size = 1 × 1 × 1 mm, 160 slices, sagittal acquisition). A field map was acquired after the functional scans using a double-echo gradient echo sequence (TE 1/2 = 4.92/7.38 msec, TR = 520 msec, flip angle = 60°, FOV = 220 mm, 48 slices, 2.5-mm slice thickness).

Data preprocessing was performed with the SPM12 software package (Wellcome Department of Imaging Neuroscience: www.fil.ion.ucl.ac.uk/spm) implemented in MATLAB 2023a. The functional images were field-map corrected, realigned, reoriented to the AC–PC line, and spatially normalized to SPM's Montreal Neurological Institute (MNI) EPI template. The normalized images were resampled to 2.5-mm isotropic voxels (thereby interpolating across the interslice gap) and smoothed with a 6-mm Gaussian kernel. The functional data from the different test blocks were concatenated using the *spm_fmri_concatenate.m* function before entry into participant-wise general linear models. Anatomical images were normalized to SPM's MNI T1 template.

### MRI Analyses

For each participant, we estimated trial-wise BOLD activity from the test phase using a single-trial general linear model that implemented the least squares all approach (Abdulrahman & Henson, 2016; Mumford, Turner, Ashby, & Poldrack, 2012). Each test event was modeled as a separate event of interest, with event-related neural activity modeled with a 4-sec duration boxcar onsetting simultaneously with the test image. Six motion regressors and four constants modeling the mean BOLD signal in the test blocks were included as covariates of no interest.

We employed the same hippocampal and left AG ROIs as those employed in Hou, Hill, Pezanko, and colleagues (2025). The hippocampal ROI was defined as all voxels falling within the 5-mm radius of the peak precision effect identified by Hou, Hill, Pezanko, and colleagues (2025; MNI coordinates: 35, −20, −18). For the left AG, we employed a combined ROI based on the peak precision effects reported by Korkki and colleagues (2023) and Richter and colleagues (2016;MNI coordinates: −54, −66, 33 and −54, −54, 33). The AG ROI comprised all voxels falling within a 5-mm radius of each peak. Trial-wise parameter estimates were extracted by averaging over all the voxels in each ROI.

In light of our prior findings (see Introduction) that trial-wise BOLD activity in both the hippocampus and left AG was predictive of memory precision, we conducted LME analyses to examine the relationships between trial-wise BOLD activity in these ROIs and the precision of memory judgments. If an ROI demonstrated sensitivity to trial-wise variability in precision, it was employed in follow-up analyses to examine whether the relationship was moderated by the inclusion of trial-wise fixation metrics as an additional predictor.

### Relationships among Memory Precision, Fixation Metrics, and Neural Activity

We employed LME analyses to examine whether fixation metrics predicted memory precision and whether either metric could account for variance in memory precision estimates beyond that accounted for by trial-wise BOLD activity. Additional analyses were conducted to assess associations between the fixation metrics and fMRI BOLD activity.

Statistical analyses were conducted with R software (R Core Team, 2018). For the pairwise *t* tests conducted at each time bin, the significance level was set at *p* < .05 after FDR correction. For all other analyses, significance levels were set at *p* < .05 after family-wise correction for multiple comparisons using the Bonferroni procedure. Note that we did not include random slopes in our LME models because their inclusion led to singular fits in most models. For models that did not exhibit singular fits when random slopes were included, the results were essentially unchanged from the models where slopes were modeled as fixed effects. Similarly, LME results were essentially unchanged when image identity was included as an additional trial-wise random intercept.

For all the LME models, we report marginal *R*^2^ and conditional *R*^2^ as estimates of the variance explained by the fixed effects and by the combination of fixed and random effects, respectively. These values were computed using the *r2()* function from the performance package in R. For models with multiple predictors, semipartial *R*^2^ is reported for each predictor to quantify the unique variance explained by that predictor. Semipartial *R*^2^ values were calculated using the *r2beta()* function with the Nakagawa–Schielzeth–Johnson method implemented in the r2glmm package in R.

## RESULTS

### Memory Performance

Across participants, the mean Pr was 0.65 (*SD* = 0.18). The mixture model yielded a precision (kappa) value of 10.76 and a guess rate (*g*) of 0.38.

### The Effect of Retrieval Success on Fixation Precision

**Figure 2.**
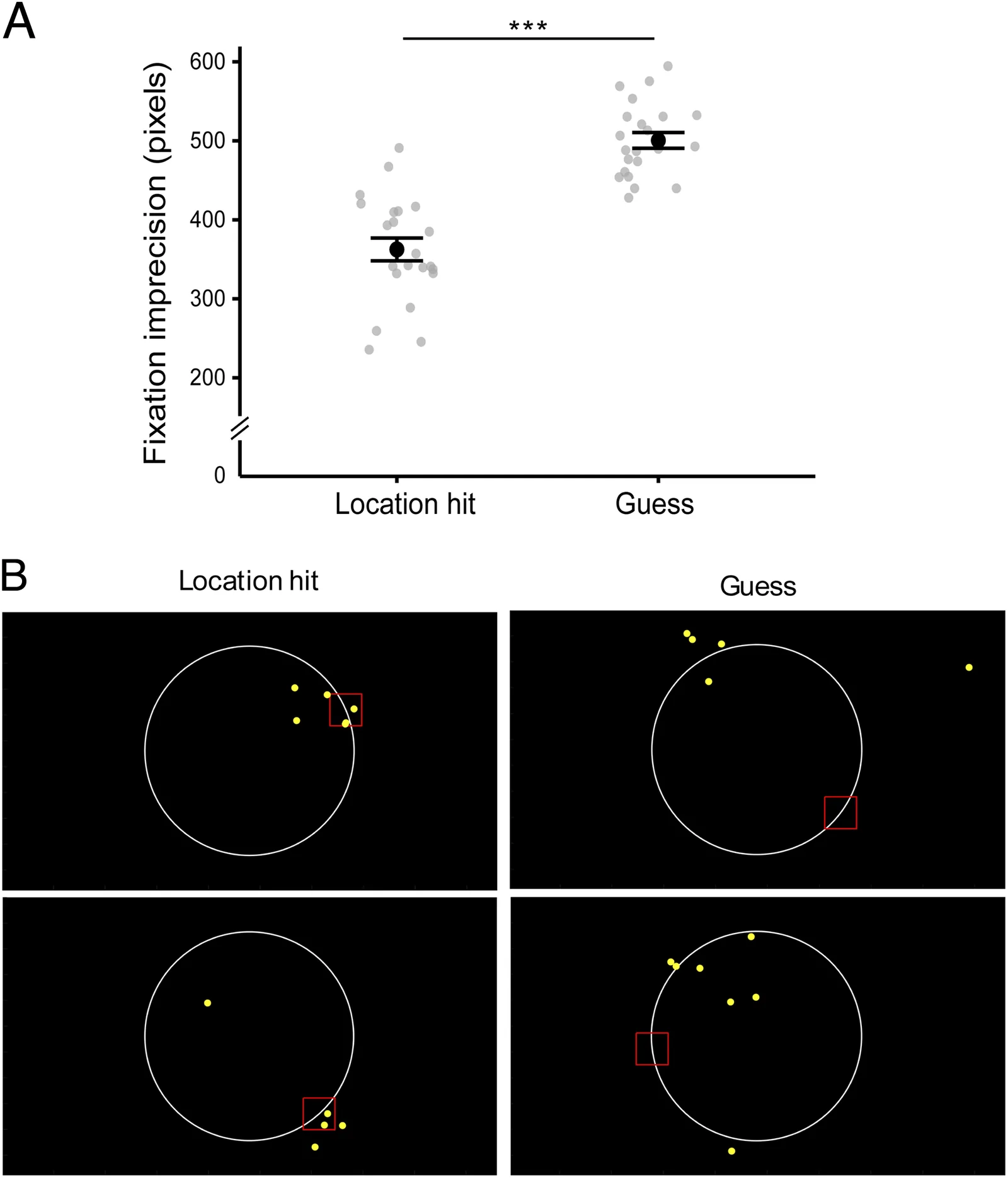
(A) Fixation precision for location hit and guess trials. Error bars represent *SEM*s. The dots represent the participant-wise mean fixation to studied location distance. ***p < .001. (B) Example trials associated with location hits and guesses from one participant. The red square represents the image's study location, and the yellow dots reflect trial-wise fixations at retrieval.

Figure 2 depicts fixation precision (as operationalized by Euclidian distance) for location hits and guesses. Location hit trials were associated with fixations that were significantly closer to the studied location, *t*(21) = 7.85, *p* < .001, Cohen's *d* = 2.38.

### Fixation Precision Predicts the Precision of Memory Judgments

**Figure 3.**
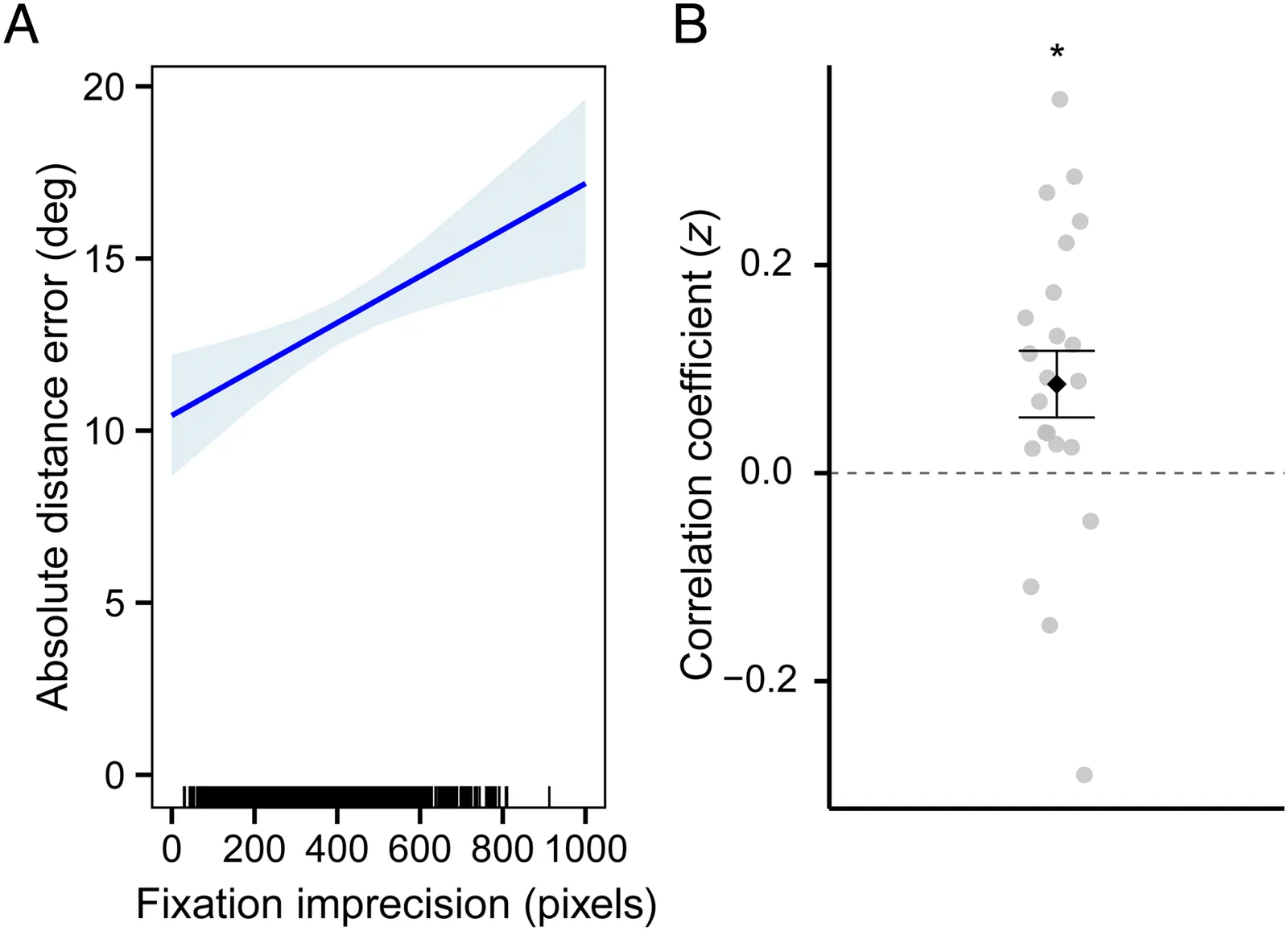
(A) Plot depicting the relationship between trial-wise fixation precision and the model fitted values of judgment precision for location hits, as derived from the LME model employing fixation precision as the predictor of judgment precision. The shaded areas reflect the 95% confidence intervals. (B) Participant-wise Pearson correlation coefficients (Fisher *z* transformed) for the relationship across trials between fixation precision and judgment precision. The error bar represents the *SEM*. deg = degrees. *p < .05.

We examined the relationship between fixation precision and the precision of the corresponding location memory judgments, focusing on location hit trials. Table 1 shows the results from the LME model that employed fixation precision as the predictor of the trial-wise precision of the corresponding memory judgment (indexed by absolute distance error). As is evident from the table, fixation precision was predictive of judgment precision; that is, fixations closer to the studied location were associated with more precise location judgments (Figure 3A).

**Table 1.** Results from a Linear Mixed Model Examining the Relationship between Fixation Precision and Trial-wise Judgment Precision for Location Hits

	*β (SE)*	*95% CI*	*df*	*t*	*p*	*Marginal R^2^*	*Conditional R^2^*
Fixation precision	0.13 (0.03)	[0.07, 0.19]	584	4.35	<.001	.017	.021

Standardized regression coefficients (β) are reported.

We also examined the pairwise correlation between the trial-wise angular error of the location judgments and fixation precision for each participant. The Fisher *z*-transformed coefficients are shown in Figure 3B. Consistent with the findings from the LME model, a one-sample *t* test revealed that the mean correlation coefficient was significantly above 0, *t*(21) = 2.67, *p* = .014, Cohen's *d* = 0.57.

### Time Series Analyses

**Figure 4.**
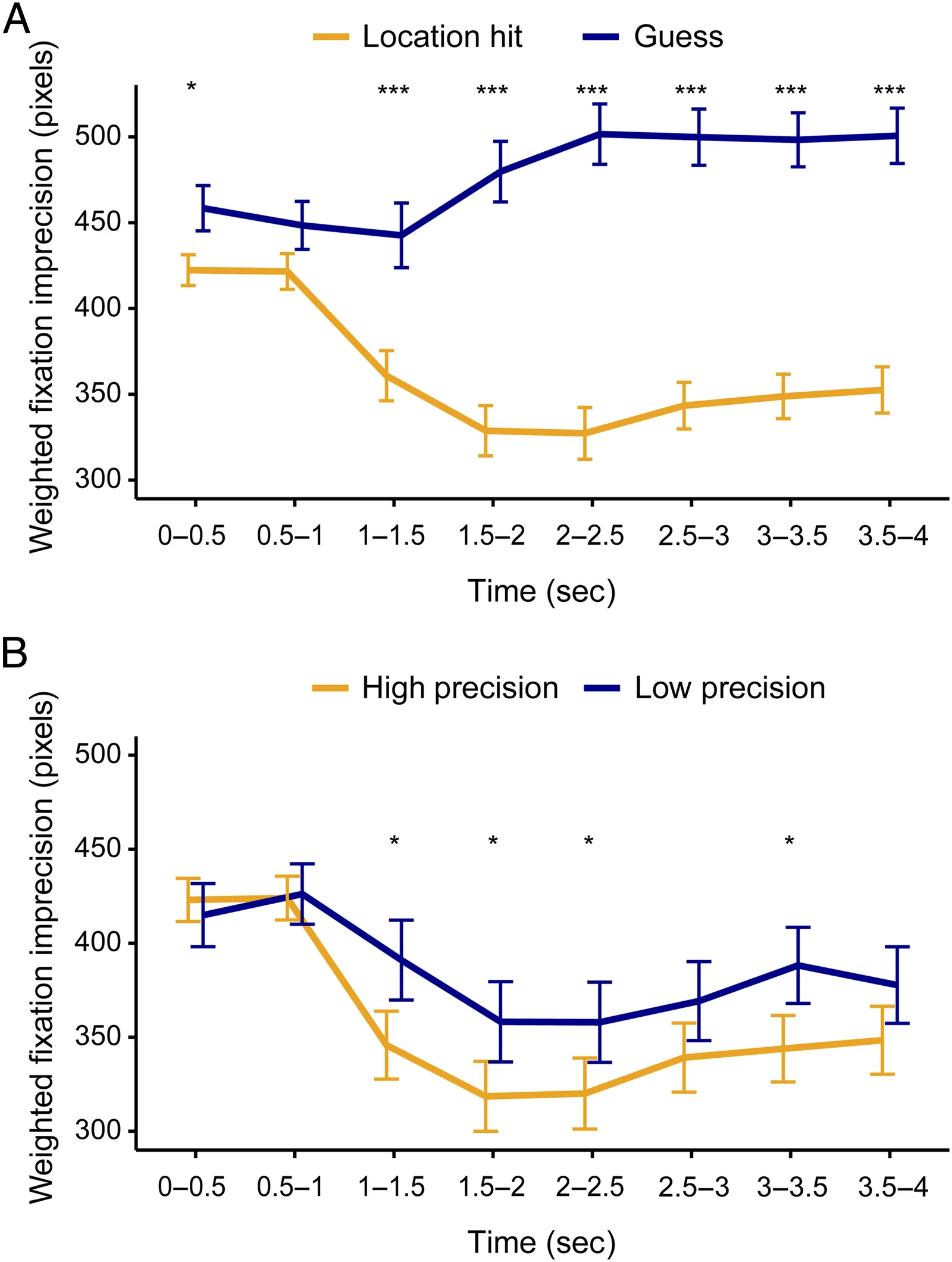
Duration-weighted fixation precision for (A) location hits versus guess trials and (B) high- versus low-precision trials. Error bars depict *SEM*s derived from the LME models. *p < .05; ***p < .001. All significant values were FDR corrected across all time bins.

Table 2 shows the results from LME models examining the effects of trial type and time bin (500-msec bins, ranging from 0 to 4 sec) on fixation precision. As is evident from the table, the model comparing location hits and guess trials revealed a significant main effect of Trial Type as well as an interaction between Trial Type and Time Bin. Follow-up analyses revealed that location hits were associated with more precise fixations than guesses in all time bins except for 0.5–1 sec (see Figure 4A).

**Table 2.** Results from LME Models Employing Trial Type and Time Bin as Predictors of Fixation Precision

	*df*	*F*	*p*	*Semipartial R^2^*	*Marginal R^2^*	*Conditional R^2^*
*Location hit vs. guess*						
Trial Type	(1, 7802)	405.53	<.001	<.001	.093	.153
Time Bin	(7, 7786)	1.56	.141	.003		
Trial Type × Time Bin	(7, 7782)	11.80	<.001	.010		

*High vs. low precision*						
Trial Type	(1, 5194)	9.46	.002	<.001	.020	.125
Time Bin	(7, 5186)	7.13	<.001	.014		
Trial Type × Time Bin	(7, 5184)	1.47	.172	.002		

As is also shown in Table 2, the model contrasting high- versus low-precision trials yielded significant main effects of trial type and time bin, indicative of more precise fixations for high- than for low-precision trials as well as for later than for earlier time bins (Figure 4B). Analyses following up the main effect of Time Bin revealed lower fixation precision in the first two bins than the later bins starting from the bin of 1.5–2 sec (*p*s < .05, FDR corrected). Despite the nonsignificant Trial Type × Time Bin interaction for memory precision, we conducted an exploratory analysis for each time bin to compare fixation precision according to trial type. Significant differences were identified from 1 to 1.5 sec postcue until 2–2.5 sec. A significant difference between the two trial types was also evident for the 3- to 3.5-sec bin (Figure 4B).

The analyses conducted on the prestimulus time bin (−0.5 to 0 sec) did not reveal a significant difference between location hits and guesses (*p* = .229) or between high- versus low-precision trials (*p* = .268).

### Fixation Pattern Similarity Analyses

#### Effects of Retrieval Success and Memory Precision on Pattern Similarity

**Figure 5.**
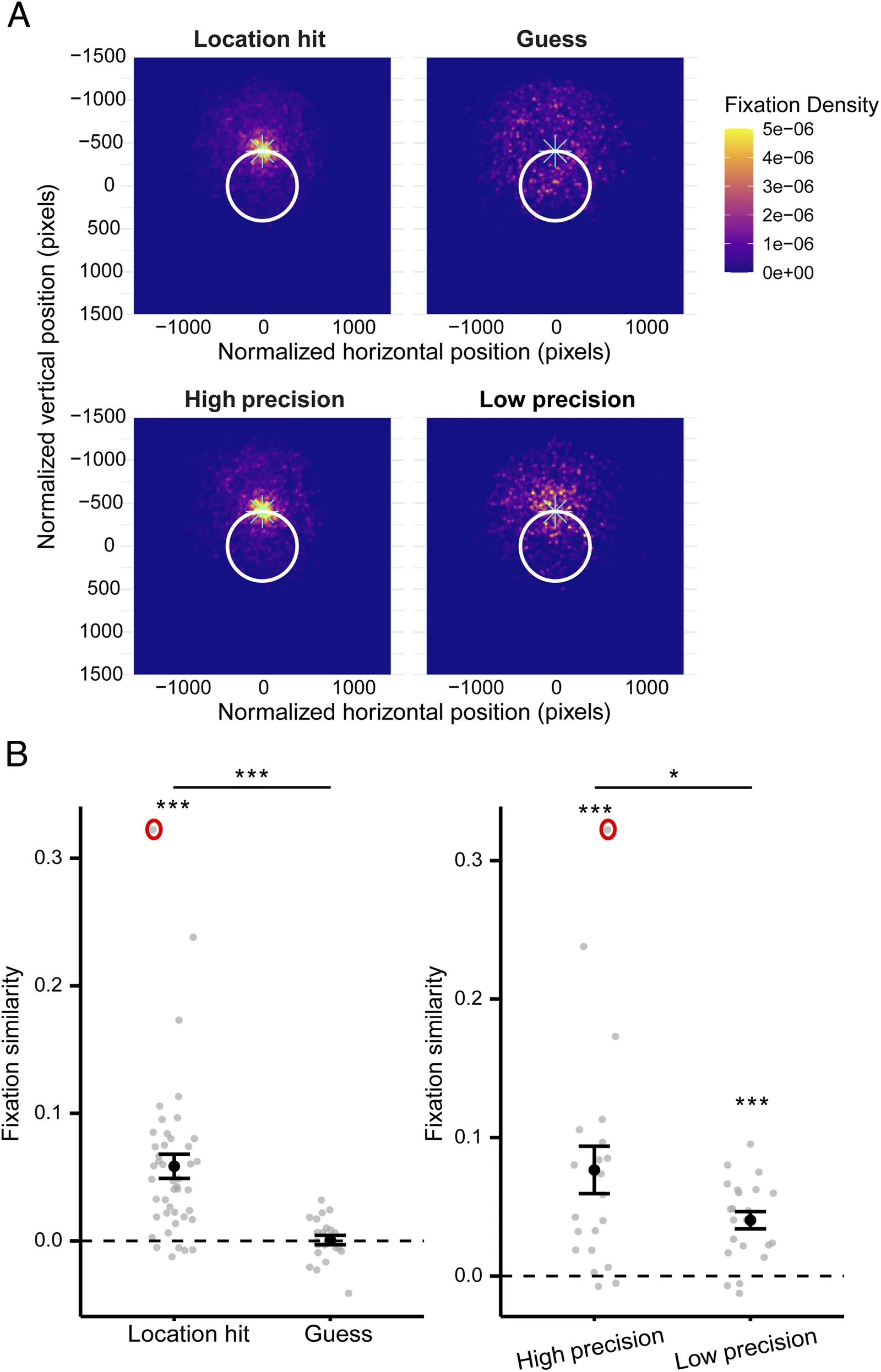
(A) Heatmaps illustrating the density of eye fixations (weighted by fixation duration) around the studied location (marked with the cyan star) for different trial types. To facilitate comparison, all studied locations were realigned to the top point of the circle, and the corresponding fixation locations were adjusted accordingly. (B) Estimates of fixation similarity of location hits and guesses. (C) Estimates of fixation similarity of high- and low-precision trials. ***p < .001; *p < .05. Error bars represent the *SEM*s. Excluding the data from the participant with the outlying data point (circled, identified as > 3 *SD*s from the mean) did not alter the results.

Figure 5A displays the fixation density maps associated with location hits and guesses, as well high- and low-precision memory judgments, aggregated across all trials and participants. To facilitate comparison, the studied location associated with all trials across participants was realigned to the same location, and their corresponding fixation locations were adjusted accordingly. As is evident from the figure, fixations were more densely distributed around the studied location for location hits than for guesses and for high-precision than low-precision trials.

A pairwise *t* test comparing the similarity metrics (see the Pattern Similarity Analysis section) between trial types revealed greater across-trial similarity for location hits than for guesses, *t*(21) = 4.44, *p* < .001, Cohen's *d* = 1.36, and for high-precision than low-precision judgments, *t*(21) = 2.77, *p* = .011, Cohen's *d* = 0.41. The similarity metrics for all trial types other than guesses were significantly greater than 0 (for guesses, *p* = .867; for other trial types, *p*s < .001; see Figure 5B and C).

#### Fixation Pattern Similarity Predicts Precision of Memory Judgments

**Figure 6.**
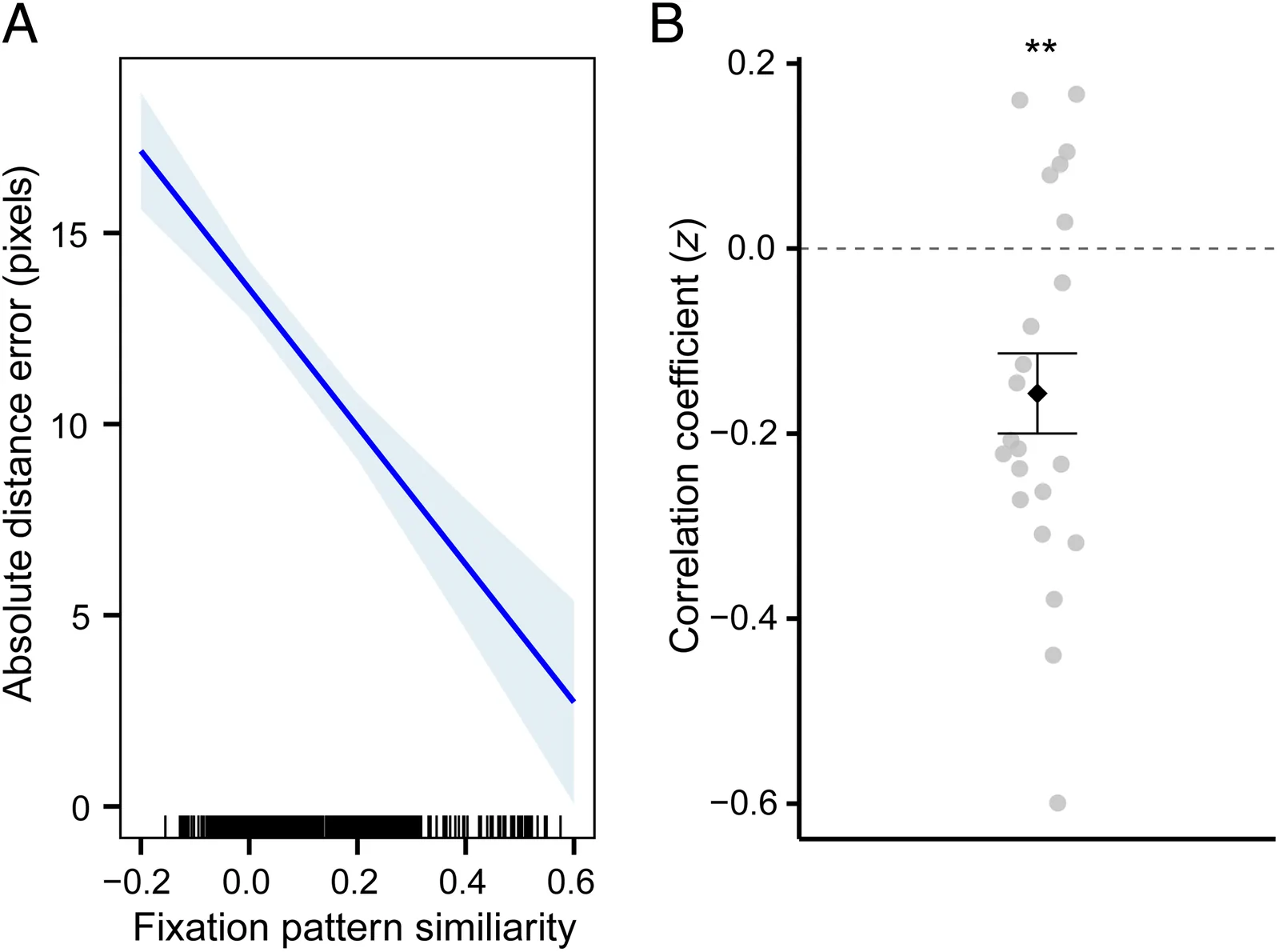
(A) Plot depicting the relationship between trial-wise fixation pattern similarity and the model fitted values of judgment precision for location hits, as derived from the LME model employing fixation pattern similarity as the predictor of judgment precision. The shaded areas reflect the 95% confidence intervals. (B) Participant-wise Pearson correlation coefficients (Fisher *z* transformed) for the relationship across trials between fixation pattern similarity and judgment precision. The error bar represents the *SEM*. **p < .01.

We went on to examine whether across-trial fixation similarity was predictive of the precision of location hits. As is shown in Table 3, the relationship between fixation similarity and memory precision was significant, with greater similarity associated with lower distance error and thus higher memory precision (see Figure 6A). In a complementary analysis, participant-level pairwise correlations were computed between the trial-wise metrics of absolute distance error and fixation pattern similarity, and the mean Fisher z-transformed correlation coefficients are shown in Figure 6B. A one-sample *t* test revealed that the mean correlation coefficient was significantly below 0, *t*(21) = 3.61, *p* = .002, Cohen's *d* = 0.77, consistent with the findings from the LME analysis.

**Table 3.** Results from Linear Mixed Models Employing Fixation Similarity as a Predictor of Trial-wise Memory Precision

	*β (SE)*	*95% CI*	*df*	*t*	*p*	*Marginal R^2^*	*Conditional R^2^*
Fixation similarity	−0.23 (0.03)	[−0.29, −0.16]	134	7.11	<.001	.051	.053

Standardized regression coefficients (β) are reported.

#### Control Analysis: Fixations in Reference to the Judged Location

To determine whether the findings reported above merely reflected preparation for the upcoming location judgment rather than reflecting retrieved memory content, we examined the mean Euclidean distance of all fixations relative to the judged location as a function of trial type. The fixation to judged location distance was significantly closer for location hits (*M* = 355, *SD* = 74) than guess trials (*M* = 408, *SD* = 68), *t*(21) = 3.43, *p* = .002, Cohen's *d* = 0.73. There was, however, no significant difference between high-precision (*M* = 357, *SD* = 82) and low-precision (*M* = 352, *SD* = 68) trials, *p* = .679.

We also analyzed the similarity of the fixation patterns associated with different trial types relative to the judged location (see Supplemental Figure S1 for the heatmaps illustrating the density of eye fixations around the judged location for different trial types). Pairwise *t* tests revealed that across-trial similarity was higher for location hits than for guesses, *t*(21) = 2.95, *p* = .008, Cohen's *d* = 1.15. By contrast, similarity did not differ between high- and low-precision judgments (*p* = .266). The similarity metrics for all trial types other than guesses were significantly greater than 0 (for guesses, *p* = .121; for other trial types, *p*s < .003).

#### Hippocampal BOLD Signals Predict Precision of Memory Judgments

**Figure 7.**
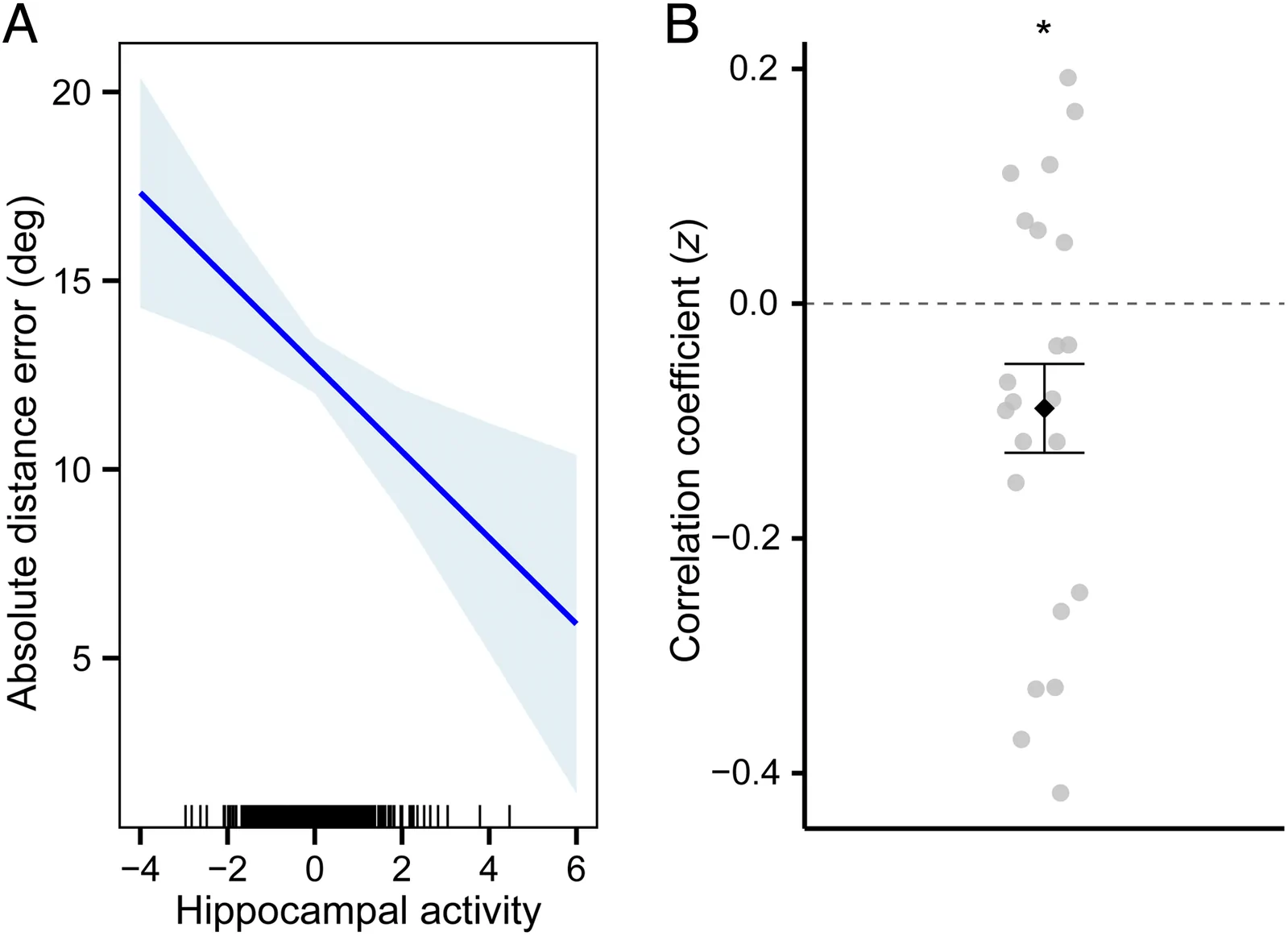
(A) Plot depicting the relationship between trial-wise hippocampal BOLD activity and the model fitted values of memory precision for location hits, as derived from the LME model employing hippocampal activity as the predictor of precision. The shaded areas reflect the 95% confidence intervals. (B) Participant-wise Pearson correlation coefficients (Fisher *z* transformed) for the relationship across trials between hippocampal activity and judgment precision. The error bar represents the *SEM*. deg = degrees. *p < .05.

In light of prior findings that fMRI BOLD activity in the hippocampus and the left AG independently predicted trial-wise memory precision (Hou, Hill, Pezanko, et al., 2025), we examined the relationships between fixation precision, precision of location judgments, and neural activity in these regions. As shown in Table 4, trial-wise BOLD activity in the hippocampus (see Figure 7A) was predictive of the precision of location judgments. We went on to conduct a pairwise correlation analysis between hippocampal BOLD activity and judgment precision across participants. As is illustrated in Figure 7B, the mean pairwise correlation coefficient across participants was significantly below 0, *t*(21) = 2.36, *p* = .028, Cohen's *d* = 0.50, consistent with the findings from the LME analysis. By contrast, the association between trial-wise AG activity and judgment precision only approached significance. We therefore focused on hippocampal activity in the subsequent analyses.

**Table 4.** Results from Linear Mixed Models Examining the Relationships between BOLD Activity in the Hippocampal and AG ROIs and Memory Precision

	*β (SE)*	*95% CI*	*df*	*t*	*p*	*Marginal R^2^*	*Conditional R^2^*
Hippocampus	−0.09 (0.03)	[−0.15, −0.03]	1161	3.04	.002	.008	.024
AG	−0.06 (0.03)	[−0.11, 0.00]	1164	1.90	.058	.003	.021

Standardized regression coefficients (β) are reported.

#### Relationships Among Memory Precision, Fixation Metrics, and Hippocampal Activity

Finally, we assessed whether eye fixation metrics and hippocampal BOLD activity independently predicted judgment precision. As is evident in Table 5, when both fixation precision and fixation pattern similarity were included in the same model as predictors of the precision of location judgments, only similarity remained significant. As is also shown in the table, each fixation metric independently predicted judgment precision when hippocampal activity was included in the model. Moreover, both fixation pattern similarity and hippocampal activity remained significant predictors after controlling for fixation precision.

**Table 5.** Results from Linear Mixed Models Employing Fixation Precision, Fixation Pattern Similarity, and Neural Activity in the Hippocampus as Predictors of Trial-wise Memory Precision

	*β (SE)*	*95% CI*	*df*	*t*	*p*	*Semipartial R^2^*	*Marginal R^2^*	*Conditional R^2^*
*Fixation precision + fixation pattern similarity*								
Fixation precision	−0.00 (0.04)	[−0.08, 0.08]	911	0.02	.984	<.001	.051	.053
Fixation pattern similarity	−0.23 (0.04)	[−0.30, −0.15]	238	5.67	<.001	.033		

*Fixation precision + hippocampus*								
Fixation precision	0.13 (0.03)	[0.07, 0.18]	553	4.24	<.001	.016	.024	.028
Hippocampus	−0.08 (0.03)	[−0.14, −0.03]	1100	2.83	.005	.007		

*Fixation pattern similarity + hippocampus*								
Fixation pattern similarity	−0.22 (0.03)	[−0.28, −0.16]	973	7.11	<.001	.049	.058	.058^a^
Hippocampus	−0.09 (0.03)	[−0.15, −0.03]	973	2.89	.004	.009		

*Fixation pattern similarity + fixation precision + hippocampus*								
Fixation pattern similarity	−0.22 (0.04)	[−0.30, −0.15]	972	5.70	<.001	.032	.058	.058^a^
Fixation precision	−0.00 (0.04)	[−0.08, 0.07]	972	0.11	.916	<.001		
Hippocampus	−0.09 (0.03)	[−0.15, −0.03]	972	2.89	.004	.009		

Standardized regression coefficients (β) are reported.

^a^ The variance accounted for by the random effect was negligible as the model resulted in a singular fit.

Additional LME analyses revealed that fixation pattern similarity was significantly predictive of fixation precision (β = −0.61, *SE* = 0.03, *t* = 21.24, *p* < .001, marginal *R*^2^ = .375, conditional *R*^2^ = .402). However, hippocampal activity did not predict either fixation metric (*p*s > .104).

## DISCUSSION

The present study examined the relationship at the time of retrieval between eye fixations and memory precision for the spatial location of studied object images. We identified robust associations between the “precision” of incidental fixations occurring before an overt memory judgment and both the success and precision of location memories. The associations took the form of fixations that were closer to the studied location for memory judgments reflecting successful location retrieval than for guesses (a retrieval success effect) and for judgments associated with high versus low precision (a memory precision effect). In addition, across-trial similarity between fixation patterns was higher for memory judgments associated with successful location retrieval than for guess trials and for high- than low-precision trials. Time series analyses revealed that the effect of retrieval success on fixation precision persisted for almost the entire recall phase. The effects of memory precision emerged around 1–1.5 sec after the onset of the cue. Finally, fixation precision and pattern similarity each predicted trial-wise memory precision independently of hippocampal fMRI BOLD activity. Below, we discuss these findings in more detail.

Consistent with prior studies (Schmidig et al., 2025; Büchel et al., 2024; Johansson & Johansson, 2014; Hannula & Ranganath, 2009), we observed higher fixation precision before overt memory judgments based on retrieved location information compared to judgments associated with guesses. More importantly, fixation precision tracked the precision of overt location judgments on a trial-wise basis. That is, eye fixations were closer to the studied location before the retrieval of precise rather than less precise location memories. These findings extend prior work (e.g., Schmidig et al., 2025; Büchel et al., 2024) by indicating that eye fixations are predictive not only of the likelihood of successful retrieval but also of the fidelity of the retrieved mnemonic content.

The retrieval success effect on fixation precision persisted across nearly the entire recall phase. By contrast, a precision effect emerged around 1–1.5 sec after the image onset. These results suggest that the sensitivity of fixations to memory content is present relatively early during recall and remains stable thereafter. The reason for the latency difference between the two effects is unclear. One possibility is that the experimental design did not provide sufficient support for participants to accurately fixate on the studied location during the early stages of recall. As is evident from Figure 1, the test image was presented in the absence of the reference circle for the first second of the recall phase. Consequently, participants would have been unable to use the circle as a fixation guide until sometime after the onset of the retrieval cue.

Alternatively, the longer latency of the precision effect might indicate that the processes supporting the precision of a memory operate more slowly than those supporting its initial retrieval. It should be noted, however, that the low temporal resolution of these analyses (500-msec time bins), combined with the limited number of high- and low-precision trials contributing to each bin, prevented a detailed characterization of the time course of these effects. Some information about the timing of the retrieval processes associated with differing levels of memory precision is available from the findings of two prior ERP studies (Ladyka-Wojcik, Schmidt, Cooper, & Ritchey, 2025; Murray, Howie, & Donaldson, 2015). In both cases, memory precision was associated with modulation of the amplitude of the so-called “left parietal effect,” a retrieval-related ERP modulation that is typically observed between about 500–800 msec after cue onset (for a recent review, see Kwon, Rugg, Wiegand, Curran, & Morcom, 2023). It is unclear how the present findings regarding the precision of eye fixations might relate to these ERP findings. Future studies with greater sensitivity and temporal resolution are needed to better characterize the temporal dynamics of retrieval-related fixations and to examine their relationship with corresponding electrophysiological effects.

Although fixation patterns were randomly distributed across guess trials, they were significantly correlated across trials on which participants successfully retrieved location information, and these correlations increased with memory precision. These findings suggest that retrieval of high-fidelity location information is associated with a systematic and relatively stereotypical spatial distribution of fixations. This likely reflects the tendency for fixations to be more consistently distributed in reference to the studied location as the precision of the memory driving them increases. By contrast, fixation dispersion—the standard deviation of within-trial fixation locations—did not show an effect of memory precision (see Supplemental results). Despite the strong across-participant correlation between fixation pattern similarity and fixation precision (*r* = .88), when both metrics were employed in the same model as predictors of the precision of overt memory judgments, pattern similarity remained as a significant predictor. Although it remains to be established whether these findings generalize to memory features other than location, our results highlight the importance of examining the spatial distribution of fixations when assessing the relationship between eye movements and memory retrieval, rather than relying solely on a univariate metric such as mean Euclidean distance.

The present findings are consistent with the proposal that eye fixations might serve as an indirect index of memory retrieval (Schmidig et al., 2025; Kragel & Voss, 2022; Ryan & Shen, 2020; Hannula et al., 2010). However, to employ fixations as a marker of retrieval, it is necessary to demonstrate that they are selectively associated with memory judgments that are specifically reflective of successful memory retrieval. To address this question, we compared fixation precision and pattern similarity relative to the judged rather than the studied location. Fixations were closer to the judged location for location hits than for guess trials, and fixation patterns associated with location hit trials were consistently clustered around the judged location, whereas guesses were associated with more diffusely distributed patterns. Thus, gaze patterns did not track judged location when there was an apparent failure to retrieve location information (this null finding is especially notable given that the use of a sample-wide cutoff raises the possibility that, in some participants, a proportion of the trials were miscategorized as guesses rather than location hits). That is, the findings indicate that eye fixations were selectively predictive of judged location only when retrieval was successful.

We also examined the associations between precision-related fixations, neural activity, and the precision of overt memory judgments. This analysis was motivated by our prior findings from a data set that included the current sample as a subsample (Hou, Hill, Pezanko, et al., 2025), where it was reported that fMRI BOLD signals in the hippocampus and left AG were each predictive of memory precision. In the reduced data set available here, only hippocampal activity tracked memory precision, consistent with the proposal that this structure contributes to the encoding and reinstatement of high-precision mnemonic information (Ekstrom & Yonelinas, 2020). We did not identify a relationship between AG activity and memory precision. This null finding likely reflects reduced power because of the smaller sample size of the present study than that of the parent study. It is worth noting, however, that in a subregion of the AG ROI employed by Hou, Hill, Aktas, and colleagues (2025), we were able to identify a significant relationship between AG activity and judgment precision (Richter ROI, β = −0.08, *SE* = 0.03, *t* = 2.72, *p* = .007).

Although both fixation precision and pattern similarity were predictive of the precision of the subsequent location judgment, neither variable was significantly correlated with hippocampal activity. Moreover, when included in the same model, the fixation metrics and hippocampal activity independently predicted judgment precision. Among other possible explanations, this finding might indicate that some neural variable other than univariate fMRI BOLD activity reflects the contribution of the hippocampus to fixation precision and pattern similarity metrics (e.g., a quite different finding might have emerged had we employed electrophysiological methods to directly sample hippocampal neural activity with high temporal resolution; cf. Kragel & Voss, 2022). Alternately, it might be that the fixation precision and pattern similarity metrics reflect the additional influence of a nonhippocampal mechanism. For example, although dependent on hippocampal retrieval processing, the metrics might also depend on other factors, such as the efficacy or precision of oculomotor control, that overshadow the contribution of the hippocampus at the trial-wise level.

### Limitations

The present study has several limitations. First, we only assessed location memory, leaving it unresolved whether the relationships between eye fixations and memory precision identified here extend to other contextual features. Second, the absence of trial-wise subjective reports such as confidence judgments precluded a full characterization of the memory signals supporting precision. Third, the experimental design was not optimized to characterize the time course of the relationship between fixations and memory precision. Finally, the low spatial resolution of the fMRI data precluded the opportunity to investigate relationships between precision-related eye movements and fMRI BOLD activity at the level of hippocampal subfields. These limitations can be addressed in future research.

### Conclusion

The present findings indicate that eye fixations are closer to and more tightly clustered around a studied location when a location memory was retrieved with high rather than low precision. Thus, the findings are consistent with the proposal that eye fixations track the fidelity of retrieved mnemonic content.

## Acknowledgments

We thank our experimental participants for volunteering their time.

Corresponding author: Mingzhu Hou, Center for Vital Longevity, 1600 Viceroy Drive, Suite 800, Dallas, TX 75235, e-mail: mingzhu.hou@utdallas.edu.

## Data Availability Statement

The data that support the findings of this study are available from the authors on request subsequent to a formal data sharing agreement. The software, functions, and syntax of the models used to generate the results are specified in the Methods section. Supplemental Material can be accessed on this article’s homepage: https://doi.org/10.1162/JOCN.a.2627.

## Author Contributions

Mingzhu Hou: Conceptualization; Data curation; Formal analysis; Investigation; Methodology; Software; Validation; Visualization; Writing—Original draft; Writing—Review & editing. Luke R. Pezanko: Investigation; Writing—Review & editing. Sabina Srokova: Methodology; Writing—Review & editing. Paul F. Hill: Writing—Review & editing. Arne D. Ekstrom: Funding acquisition; Writing—Review & editing. Michael D. Rugg: Conceptualization; Funding acquisition; Methodology; Resources; Supervision; Writing—Original draft; Writing—Review & editing.

## Funding Information

This work was supported by the National Institute of Neurological Disorders and Stroke (grant R01NS114913).

## Diversity in Citation Practices

Retrospective analysis of the citations in every article published in this journal from 2010 to 2021 reveals a persistent pattern of gender imbalance: Although the proportions of authorship teams (categorized by estimated gender identification of first author/last author) publishing in the *Journal of Cognitive Neuroscience* (*JoCN*) during this period were M(an)/M = .407, W(oman)/M = .32, M/W = .115, and W/W = .159, the comparable proportions for the articles that these authorship teams cited were M/M = .549, W/M = .257, M/W = .109, and W/W = .085 (Postle and Fulvio, *JoCN*, 34:1, pp. 1–3). Consequently, *JoCN* encourages all authors to consider gender balance explicitly when selecting which articles to cite and gives them the opportunity to report their article's gender citation balance.

## Supplementary Material


