# Supplementary material for: Retrieval-related Eye Movements Are Predictive of Memory Precision
Source: J Cogn Neurosci. 2026 Oct 1;38(10):1922–36. doi: 10.1162/JOCN.a.2627 (PMC13569477; doi:10.1162/JOCN.a.2627)
Supplement: Supplementary file 1 [file jocn-38-10-1922-s001.docx]

**Retrieval-related eye movements are predictive of memory precision**

Mingzhu Hou, Luke R. Pezanko, Sabina Srokova, Paul F. Hill, Arne D. Ekstrom and Michael D. Rugg

Supplemental materials

Heatmaps illustrating the density of fixations in the control analysis

Supplemental Figure 1 shows the heatmaps reflecting the density of fixations around the judged location associated with different trial types. As is evident from this figure, fixations were more tightly clustered around the response location for location hits than for guess trials. By contrast, high and low-precision trials showed similar fixation patterns.

Supplemental Figure 1. Heatmaps illustrating the density of eye fixations (weighted by fixation duration) around the judged location (marked as the cyan star) for different trial types. To facilitate comparison, all response locations were realigned to the top point of the circle, and the corresponding fixation locations were adjusted accordingly.

Dispersion analysis

We computed trial-wise fixation dispersion based on the fixation maps employed in the pattern similarity analysis (see main text). Following the procedure employed in Wynn et al. (2025), fixation dispersion was calculated as the mean standard deviation of X and Y fixation coordinates. Across participants, we found lower dispersion for location hits (M = 128.30, sd = 24.61) than guesses [M = 138.08, sd = 29.43, t(21) = 2.42, p = 0.025]. By contrast, there was no significant difference between high (M = 125.97, sd = 22.98) and low precision trials [M = 133.54, sd = 32.43, t(21) = 1.42, p = 0.169]. In the LME analysis, trial-wise fixation dispersion did not significantly predict the absolute distance error of location hits (β = 0.05, SE = 0.03, t = 1.61, p = 0.107).

Reference

Wynn, J. S., Khosla, A., Kacollja, A., Shahmiri, E., Anderson, N. D., Shen, K., & Ryan, J. D. (2025). Decoding memory function through naturalistic gaze patterns. Proceedings of the National Academy of Sciences, 122, e2505879122. DOI: https://doi.org/10.1073/pnas.2505879122
